# Change in Programmed Death-1 and Inducible Costimulator Expression in Patients whit Acute Myeloid Leukemia Following Chemotherapy and Its Cytogenetic Abnormalities: Change in PD-1 and ICOS Expression in Patients whit AML

**DOI:** 10.31661/gmj.v11i.2394

**Published:** 2022-12-31

**Authors:** Mahdiyar Iravani Saadi, Maryam Ahmadyan, Heeva Jalali, Nasrin Noshadi, Mitra Moradi, Fatemeh Mardani Valandani, Nadiya Kheradmand, Hossain Ali Rostamipour, Fakhroddin Hosseini, Amir Ali Hamidieh, Abolfazl Khalafi-Nezhad

**Affiliations:** ^1^ Pediatric Cell and Gene Therapy Research Center, Gene, Cell and TissueResearch Institute,Tehran University of Medical Sciences, Tehran, Iran; ^2^ Hematology Research Center, Shiraz University of Medical Sciences, Shiraz, Iran; ^3^ Department of Animal Science, Faculty of Agriculture, University of Kurdistan, Sanandaj, Iran; ^4^ Department of Internal Medicine, Jahrom University of Medical Sciences, Jahrom, Iran; ^5^ Hematology, Oncology and Bone Marrow Transplantation Department, Shiraz University of Medical Sciences, Shiraz, Iran

**Keywords:** Acute Myeloid Leukemia, Programmed Death, Inducible Costimulator, Induction Therapy, Mononuclear Cells

## Abstract

**Background:**

Programmed death-1 (PD-1) and inducible costimulator (ICOS) are immune checkpoint receptors participating in tumor immune evasion, which counters the activation signal provided through the T-cell receptor ligation. This study aimed to investigate the relationship between the expression of PD-1 and ICOS on mononuclear cells (MNCs) isolated from the peripheral blood of acute myeloid leukemia (AML) patients and their response to induction chemotherapy.

**Materials and Methods:**

Peripheral blood samples (5 cc) were collected from 56 AML patients at first diagnosis before and after the induction therapy regimen for AML. PD-1 and ICOS expression were analyzed in all patients before and after the standard induction therapy regimen.

**Results:**

The expression of PD-1 and ICOS significantly decreased (66.7 and 16.3 fold, respectively) in AML patients following chemotherapy compared to its baseline value (P=0.01 and P=0.001, respectively). The expressions of PD-1 and ICOS were significantly different between favorable and poor risk groups.

**Conclusions:**

Lower PD-1 and ICOS expressions on the surface of MNCs before induction therapy were associated with a better response to treatments. In addition, PD-1 and ICOS expression on MNCs decreased after induction therapy.

## Introduction

Acute myeloid leukemia (AML) is the most common form of acute leukemia in adults [1]. The principal feature of AML is its variable responses to therapy [2]. Also, new studies have revealed that chemical drug resistance rather than treatment-related mortality is the major cause of treatment failure in AML [3]. The general therapeutic strategy in patients with AML is intensive induction chemotherapy followed by appropriate post-remission therapy after achieving complete remission [4]. The mainstay of initial induction therapy for AML is cytarabine in combination with an anthracycline (i.e., daunorubicin or idarubicin) [5]. Although short-term complete remission is achieved after standard chemotherapy in most cases, long-term remission is obtained in less than 50% of the patients [6]. Hence, some efforts have been made to target the immune system as a novel therapeutic modality for treating AML because of the proven role of the immune system in the control of AML [7]. These efforts include treatment options such as multispecific antibody (Ab) constructs and treatments targeting immune checkpoint inhibitors [7][8]. PD-1, an immune checkpoint receptor inducibly expressed on B-cells, T-cells, and activated monocytes, belongs to the immunoglobulin (Ig) superfamily [9]. Recently, the crucial role of PD-1 signaling has been identified in regulating peripheral tolerance and autoimmunity by inducing a coinhibitory signal in activated T-cells leading to T-cell apoptosis and functional exhaustion [10]. Recent evidence indicates that PD ligand 1 (PD-L1) expression may be related to tumor growth promotion and apoptosis induction [11]. Also, it has been shown that blocking the PD-L1/PD-1 pathway using anti-PD-L1 or anti-PD-1 monoclonal Ab results in the inhibition of tumor growth and boosting of antitumor immunity [11]. Also, it has been proposed that the PD-1/PD-L1 mechanism plays a role in the interaction of AML blasts with the immune system in mouse models [12]. Increased expression of PD-L1 was identified in blast cells from AML patients exposed to immune response, pathogens, and sometimes upon relapse, and thus it was considered as an immune escape molecule [13]. However, in another study, it was concluded that PD-1 was not related to the survival of AML patients [14]. According to several studies, ICOS appears to play a role in solid organ graft rejection. ICOS inhibition improved cardiac transplant survival, according to Harada et al. while liver transplant survival was improved in another trial [15]. Fewer studies have looked at the involvement of the ICOS pathway in bone marrow (BM) transplantation, while one study utilized non-irradiated parent-into-F1 models. Burlion et al. investigated that ICOS inhibition decreased T helper 2 (Th2)-mediated chronic graft-versus-host disease (GVHD) but increased Th1-mediated acute GVHD [16]. In additional trials, ICOS blocking has also been beneficial for GVHD prevention, therapy, and allogeneic BM graft promotion [16]. Hence, ICOS expression best characterizes inflammatory effector T-cells 26, imply that the ICOS pathway could be a promising therapeutic target [15]. Profiling the surface expression of PD-1 on mononuclear cells (MNCs) isolated from peripheral blood (PB) of AML patients may represent valuable data anticipating the patient’s response to the standard induction chemotherapy regimen. Hence, this study aimed to assess the relationship between the expression of PD-1 on MNCs isolated from the PB of AML patients and their response to induction chemotherapy.

## Materials and Methods

**Table:** Table1. The Primers and PCR Conditions

**Genes**	**Primer sequences (5'-¬¬¬¬¬¬¬¬¬¬¬¬¬¬¬>3') **	**Thermocycling condition**
***PD-1***	F: CTTAGACTCCCCAGACAGG	
	R: GCGTTGTCCCCTTCGGT	95 °C for 2 min, 40 cycles of 95 °C for 30 sec, 60 °C for 20 sec, and 70 °C for 30 sec
***ICOS***	F: TTGAACACTGAACGCGAGGA	
	R: GCAGAACCATTGATTTCTCCTGT	
**GAPDH**	F: GGACTCATGACCACAGTCCA	95 °C for 2 min, 40 cycles of 95 °C for 30 sec, 57.5 °C for 20 sec, and 70 °C for 30 sec
	R: CCAGTAGAGGCAGGGATGAT	

**PD-1:** Programmed death-1; **ICOS:** Inducible costimulator; **GAPDH:** Glyceraldehyde 3-phosphate dehydrogenase

### Patients and Design

Fifty-six newly diagnosed adult AML patients who were referred to Namazi Hospital (Shiraz, Iran) were selected. PB samples (5 cc) were collected from the patients before starting treatment. WHO criteria were used to diagnose and evaluate the morphology and cytochemistry of BM samples collected from the patients. Complete blood count, blast percentage, and hemoglobin (Hb) levels were also collected. All patients received a standard induction therapy regimen for AML consisting of daunorubicin (45 mg/m2) on days one to three, and cytarabine (100-200 mg/m2) on days one to seven, followed by high doses of a cytarabine-based consolidation phase (cytarabine 3 g/m2 every 12 hours for three days, repeated for two to three cycles) except for promyelocytic AML patients for whom ATRA was added to this regimen. A second BM examination was performed after induction therapy on days 17-21. Post-induction chemotherapy samples from PB were also collected from all patients. The isolation of MNCs from whole PB samples was performed by Ficoll density gradient centrifugation. Complete remission was considered as no evidence of extramedullary leukemia, no Auer Rods, neutrophil and platelet recoveries to 1-l09 and 100•109/l, respectively, and <5% blasts in a BM aspirate (irrespective of cellularity) [17][18].

### Determined Cytogenetic Status

The National Comprehensive Cancer Network (NCCN) guidelines were used to stratify cytogenetic status [19]. The presence of t (8;21), t (15;17), or inv/t (16), (nucleolar phosphoprotein B2) NPM1 or mutated (CCAAT/enhancer binding protein-alpha) CEBPA without FLT3-ITD was considered as a favorable risk abnormality. NCCN guidelines categorize an intermediate risk abnormality with the presence of normal cytogenetics, trisomy 8, t (9;11), t (8;21), inv (16), and t (16;16) with the cKIT mutation. A poor risk abnormality was defined by the presence of t11q23 (other than t [9;11]), del5/5q, del7/7q aberrations, t (6;9), inv3, t (3;3) aberrations, or a complex karyotype (three or more numerical and/or structural abnormalities), and normal cytogenetics with FLT3-ITD mutation.

### RNA Isolation and cDNA Synthesis

The total RNA was extracted by RNX-Plus solution (CinnaGen, Tehran, Iran). The quantity of the extracted RNA was evaluated by the NanoDrop 2000c (Thermo Scientific, Waltham, MA, USA) that measured the optical density 260/280, and the quality of the extracted RNA was assessed by running 3 μL on 1% agarose gel. The quality of RNAs was indicated by the lack of a smear on the lower part of the gel (a smear indicates RNA degradation) and by the presence of 28S ribosomal RNA twice as intense as 18S rRNA. After obtaining a good-quality total RNA, cDNA was synthesized using Prime Script RT Reagent Kit (Takara, Japan) according to the manufacturer’s guidelines.

### Real-time Polymerase Chain Reaction (PCR)

The real-time PCR using SYBR green (Applied Biosystems, USA) was performed to quantitatively analyze PD-1 and ICOS mRNA expression profiles. The glyceraldehyde 3-phosphate dehydrogenase (GAPDH) gene was used as an internal control for minor fluctuations. The PCR program and primer sequences are summarized in Table 1. The melt curve was analyzed to confirm the specificity of the reaction at the end of the program. The results for the target genes were measured as fluorescent signal intensity and normalized to the internal standard gene GAPDH.

Cycle threshold (Ct) values, which are inversely proportional to the original relative expression level of the gene of interest, were used to calculate the relative quantitation. The changes in the relative expression levels of PD-1 and ICOS mRNA were calculated by 2-ΔΔCt method, where ΔΔCt=(ΔCt [before chemotherapy]-ΔCt [after chemotherapy]) and ΔCt=(Ct [sample]-Ct [housekeeping gene]).

### Ethical Considerations

All the patients signed the written informed consent by the Helsinki protocol of 1975, and the study was approved by the Ethical Committee of Shiraz University of Medical Sciences (code: 1394-01-32-10603).

### Statistical Analysis

The data were analyzed by SPSS Statistics for Windows, version 15 (SPSS Inc., Chicago, Ill., USA). The differences in the mean expression level of PD-1 and ICOS before and after chemotherapy were compared via paired t-test. Also, the mean expression level of PD-1 and ICOS regarding laboratory data were analyzed by the chi-square test. The expression level of PD-1 and ICOS were compared among the patients according to their response to chemotherapy treatment, cytogenetic aberration, and French-American-British (FAB) subtypes by an independent t-test. A P-values less than 0.05 were considered as significant differences.

## Results

**Figure-1:**
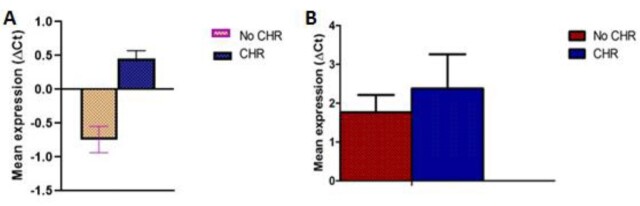
Figure 1.Changes in programmed death-1 (PD-1) (A) and inducible costimulator (ICOS) (B) expressions following chemotherapy

**Figure-2:**
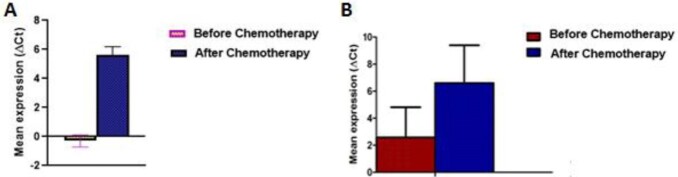
Figure 2. Programmed death-1 (PD-1) (A) and inducible costimulator (ICOS) (B) expressions in AML patients and their response to treatment

**Table:** Table2. Patient Characteristics

Variables	No. of patients (%)
Sex	
Male	30 (53.5)
Female	26 (46.5)
AML types	
With minimally differentiated	3 (5.3)
Without maturation	5 (8.9)
With maturation	26 (46.4)
Myelomonocytic leukemia	13 (22.7)
Monoblastic	9 (16)
Response rate	
CR	30 (53.5)
NCR	26 (46.5)
Cytogenetic/molecular risk	
Favorable	22 (39.3)
Intermediate	23 (41.1)
Poor	11 (19.6)

**AML:** Acute myeloid leukemia; **CR:** Complete response; **NCR:** Non-complete response

The relative mRNA expression level of PD-1 and ICOS from 56 patients (30 males and 26 females) was evaluated by real-time PCR. Patient characteristics are listed in Table 2. The median age of patients was 49 years (ranged 20-86 years). The median count of white blood cell (WBC), Hb, and platelet were 27.3×109 (ranged 1.3-147), 8.1 g/dl (ranged 3.8-14.7), and 54×109 (ranged 3-267). The mean baseline PB and BM blast percentages were 65% (ranged 18-90) and 68% (ranged 20-98), respectively.

Change in PD-1 and ICOS Expression in AML Patients Following Chemotherapy

Based on the results, the expression of PD-1 and ICOS significantly decreased (66.7, 16.3 fold) in AML patients following chemotherapy compared to its baseline value (Figure-1 and -2).

### Also, PD-1 and ICOS expression levels

were not significantly higher in AML patients who did not respond to chemotherapy than in those who responded to chemotherapy (Figure-1 and -2).

### PD-1 and ICOS Expression According to Cytogenetic Abnormalities

Delta Ct values for PD-1 and ICOS mRNA expression levels were compared among favorable (11 cases), intermediate (23 cases), and poor (22 cases) risk groups. The expression of PD-1 and ICOS were significantly different between favorable and poor risk groups (favorable vs. poor: 0.356 vs. -0.293, P=0.03, and -1.2 vs. -0.2.3, P=0.002, respectively). However, the expression of PD-1 and ICOS were not different between favorable and intermediate groups (favorable vs. intermediate: 0.365 vs. 0.492, P=0.568, and 1.2 vs. 1.1, P=0.59). The expressions of PD-1 and ICOS were not different between intermediate and poor risk groups, although the poor risk group exhibited a higher expression of PD-1 and ICOS (0.492 vs. -0.293, P=0.91, and 0.1 vs. -0.2.3, P=0.72).

### PD-1 and ICOS Expression in AML Patients and Their Response to Treatment

In the favorable risk group, the expression of PD-1 and ICOS were different in seven responders cases (Ct=-0.761 and Ct=-0.861, respectively) vs. four non-responders (Ct=2.3 Ct=1.5, respectively) to induction therapy (P=0.023 and P=0.04, respectively). No differences in PD-1 and ICOS expression were found in the complete response (CR) group (eight cases, Ct=-0.796 and Ct=-0.921, respectively) vs. the non-CR (NCR) group (14 cases, Ct=-0.147 Ct=-0.864, respectively) to the first anthracycline-based induction therapy in the poor risk group (P=0.82 and P=0.92, respectively). In the intermediate risk group, PD-1 and ICOS did not show significant differences between the CR (14 cases) and the NCR groups (nine cases) (CR vs. NCR; -0.845 vs. -0.744, P=0.969, and 0.652 vs. -0.951, P=0.72, respectively).

Expressions of PD-1 and ICOS were not significantly different among the types of AML (P=0.861). Also, there was no significant association between PD-1 and ICOS expression and cytogenetics. No significant differences were observed between mean PD-1 and ICOS expression and sex, Hb, WBC count, and blood group (P>0.05).

## Discussion

In this study, we aimed to identify the clinical role of PD-1 gene expression level in response to induction chemotherapy to find a possible prognostic marker for patient risk stratification at first diagnosis leading to the selection of a suitable treatment plan. Lower PD-1 expression before induction therapy was associated with a better response to induction therapy. In addition, PD-1 expression decreased after induction therapy.

Recently, the important role of the PD-1/PD-L1 signaling pathway in tumor immune evasion has gained widespread attention [20]. PD-1 is an immune checkpoint acting as a negative regulator of activated T-cells [20]. However, the up-regulation of surface expression and inhibitory functions of these receptors in T-cells of the tumor microenvironment leads to T-cell dysfunction and, consequently, impaired antitumor immunity [17][21]. Animal models of tumor growth have demonstrated that this immune evasion mechanism operating in cancer can be suppressed by blocking checkpoint receptors with Abs leading to antitumor immunity restoration and prevention of tumor progression [22][23]. Hence, it seems that some human tumors, including hematological malignancies, may also benefit from checkpoint inhibition with anti-PD-1 Ab. Pembrolizumab and nivolumab are anti-checkpoint Abs targeting PD-1. They have been approved for melanoma, and their efficacy for the treatment of other cancers, including hematological malignancies is currently being intensively investigated [24].

On the other hand, increased Treg frequency, which is often observed in AML patients at various stages of diagnosis and treatment, has been correlated with poor prognosis and leukemia relapse [25][26]. Also, it has been shown that the development of peripheral (inducible) Treg (pTreg) from human Th1 cells is conducted by the expression of PD-L1 in the tumor microenvironment. Therefore, the blockade of the PD-1/PD-L1 pathway in the tumor microenvironment can be correlated with the arrest of Treg-mediated immunosuppression [17][27]. Some previous studies have demonstrated the effective role of anti-PD-1 Ab therapy in refractory and relapsed Hodgkin’s lymphoma, melanoma, and advanced multiple myeloma [24][28][29]. Also, the impairment of the T-cell function caused by high PD-1 expression on CD4+ T-cells in diffuse large B-cell lymphoma has been associated with a poor prognosis. Furthermore, compared to PD-1 negative patients, reduced event-free survival and overall survival were found in patients with positive PD-1 expression within the tumor microenvironment [30]. More importantly, increased PD-1 expression in PB MNCs subsets in patients with renal cell carcinoma and the observed correlation between those expression levels in effector T-cells and the disease stage may indicate the probable role of PD-1 expression as a biomarker for predicting disease severity and prognosis [17][31].

The current study explored the relationship between PD-1 expression on the surface of MNC isolated from the PB of AML patients and their response to induction chemotherapy. Lower PD-1 expression before induction therapy was found to be associated with a better response to it. In addition, PD-1 expression in the MNCs decreased after induction therapy. Because one of the most important prognostic factors in AML patients is their response to the first induction therapy [32] and based on the obtained results of this study, it can be suggested that higher PD-1 expression in AML patients is associated with poor prognosis. Our data are in line with some previous studies concerning the relationship between the high level of PD-1 expression and poor prognosis. Muenst et al. found that PD-1-positive lymphocytes >23/mm2 in classical Hodgkin lymphoma patients were associated with poor prognosis compared to PD-1-positive cells <23/mm2 [33]. Also, PD-1 expression on the surface of CD4+ or CD8+ T-cells within follicular lymphoma was suggested as an unfavorable prediction factor for transformation, thereby leading to poor prognosis [34][35]. Furthermore, a higher number of relapses was associated with high PD-1 expression in AML patients [19]. In contrast, Yang et al. demonstrated that high PD-1 expression on the surface of CD4+ or CD8+ T-cells within follicular lymphoma did not affect on patient prognosis, while low PD-1 expression levels were correlated with poor prognosis [36]. More interestingly, Carreras et al. found that higher PD-1-positive cells in follicular lymphoma led to higher 5-year overall survival and progression-free survival [37]. The PD-1 expression on different cells with different contributions to the anticancer activity (T-cells, natural killer cells, and macrophages) in the tumor microenvironment is one of the major reasons justifying the complex relationship between PD-1 expression in the tumor environment and prognosis [30].

The detection of markers for prognostic evaluations and prediction of responses to treatment or remission rates are important for developing a suitable treatment plan in a patient’s initial diagnosis of AML. Based on our study, PD-1 can be suggested as a potential prognostic marker in AML. However, the exact role of PD-1 in AML prognosis is still unclear and must be elucidated in further studies. Also, it was found that higher PD-1 expression was associated with unfavorable courses of the disease. Recent research has found that in addition to immune system cells, myeloid leukemic cells could express costimulatory molecules such as ICOS and PD-1, which can thwart an effective anti-leukemic T-cell response [38].

In this context, the expression of B7 superfamily costimulatory molecules B7-2 (CD86) and B7-H2 (ICOSL) is important. The CD28/cytotoxic T lymphocyte antigen 4 (CTLA-4) family member inducible costimulatory ICOS is expressed on activated T lymphocytes. Furthermore, the existence of B7-2+ and/or B7-H2+ AML cell subpopulations has been linked to poor clinical outcomes such as hyperleukocytosis and limited disease-free or relapse-free survival [18][38][39]. Dolen et al. indicated that the increased expression of B7 family molecules like B7-H1 (PD-L1) and B7-DC (PD-L2) on AML cells, as well as downregulation of B7-H2 (ICOSL) on AML cells, which is linked to the immunosuppressive phenotype of AML cells, attenuation of helper T-cell responses, and promotion of regulatory T-cell differentiation, mainly through the PD1 pathway [20][40]. In addition to immune system cells, PB mononuclear cells make up a portion of leukemic blasts; therefore, alterations in CD28 and CTLA-4 gene expression could also be attributed to AML blasts. The present study had some limitations. For example, the present study did not assess prognostic parameters like overall survival.

Furthermore, follow-up time (short or long) should be considered in the interpretation of the findings of different studies. Also, only the response to induction therapy was assessed in this study, while studies of consolidation therapy with extended follow-up times may reveal different results. Therefore, future studies with extended follow-up times are needed to explore the relationship between PD-1 expression and overall survival or relapse rate.

## Conclusion

Our study revealed that PD-1 and ICOS expression before induction therapy could be associated with patients' response to induction therapy. Indeed, low PD-1 and ICOS expression levels before induction therapy were associated with favorable responses.

## Conflict of Interest

The authors had no conflict of interest to declare.

## References

[R1] Rotchanapanya W, Hokland P, Tunsing P, Owattanapanich W (2020). Clinical Outcomes Based on Measurable Residual Disease Status in
Patients with Core-Binding Factor Acute Myeloid Leukemia: A Systematic
Review and Meta-Analysis. J Pers Med.

[R2] Estey EH (2013). Acute myeloid leukemia: 2013 update on risk-stratification and
management. Am J Hematol.

[R3] Yanada M, Garcia-Manero G, Borthakur G, Ravandi F, Kantarjian H, Estey E (2008). Relapse and death during first remission in acute myeloid
leukemia. Haematologica.

[R4] Dohner H, Weisdorf DJ, Bloomfield CD (2015). Acute Myeloid Leukemia. N Engl J Med.

[R5] Gupta V, Tallman MS, Weisdorf DJ (2011). Allogeneic hematopoietic cell transplantation for adults with
acute myeloid leukemia: myths, controversies, and unknowns. Blood.

[R6] Shah A, Andersson TM, Rachet B, Bjorkholm M, Lambert PC (2013). Survival and cure of acute myeloid leukaemia in England,
1971-2006: a population-based study. Br J Haematol.

[R7] Austin R, Smyth MJ, Lane SW (2016). Harnessing the immune system in acute myeloid leukaemia. Crit Rev Oncol Hematol.

[R8] Fenaux P, Mufti GJ, Hellstrom-Lindberg E, Santini V, Gattermann N, Germing U, et al (2010). Azacitidine prolongs overall survival compared with conventional
care regimens in elderly patients with low bone marrow blast count acute
myeloid leukemia. J Clin Oncol.

[R9] Okazaki T, Honjo T (2006). The PD-1-PD-L pathway in immunological tolerance. Trends Immunol.

[R10] Francisco LM, Salinas VH, Brown KE, Vanguri VK, Freeman GJ, Kuchroo VK, et al (2009). PD-L1 regulates the development, maintenance, and function of
induced regulatory T cells. J Exp Med.

[R11] Nomi T, Sho M, Akahori T, Hamada K, Kubo A, Kanehiro H, et al (2007). Clinical significance and therapeutic potential of the programmed
death-1 ligand/programmed death-1 pathway in human pancreatic cancer. Clin Cancer Res.

[R12] Zhang L, Gajewski TF, Kline J (2009). PD-1/PD-L1 interactions inhibit antitumor immune responses in a
murine acute myeloid leukemia model. Blood.

[R13] Berthon C, Driss V, Liu J, Kuranda K, Leleu X, Jouy N, et al (2010). In acute myeloid leukemia, B7-H1 (PD-L1) protection of blasts
from cytotoxic T cells is induced by TLR ligands and interferon-gamma and
can be reversed using MEK inhibitors. Cancer Immunol Immunother.

[R14] Barsoum IB, Smallwood CA, Siemens DR, Graham CH (2014). A mechanism of hypoxia-mediated escape from adaptive immunity in
cancer cells. Cancer Res.

[R15] O’Neill NA, Zhang T, Braileanu G, Cheng X, Hershfeld A, Sun W, et al (2018). Pilot study of delayed ICOS/ICOS-L blockade with αCD40 to
modulate pathogenic alloimmunity in a primate cardiac allograft model. Transplant Direct.

[R16] Burlion A, Brunel S, Petit NY, Olive D, Marodon G (2017). Targeting the Human T-Cell Inducible COStimulator Molecule with a
Monoclonal Antibody Prevents Graft-vs-Host Disease and Preserves Graft vs
Leukemia in a Xenograft Murine Model. Front Immunol.

[R17] Begna KH, Ali W, Naseema G, Elliott MA, Al-Kali A, Litzow MR, et al (2021). Mayo Clinic experience with 1123 adults with acute myeloid
leukemia. Blood Cancer J.

[R18] De Greef GE, van Putten WL, Boogaerts M, Huijgens PC, Verdonck LF, Vellenga E, et al (2005). Criteria for defining a complete remission in acute myeloid leukaemia revisited. An analysis of patients treated in HOVON-SAKK co-operative group studies. Br J Haematol.

[R19] Schmohl JU, Nuebling T, Wild J, Kroell T, Kanz L, Salih HR, et al (2016). Expression of RANK-L and in part of PD-1 on blasts in patients
with acute myeloid leukemia correlates with prognosis. Eur J Haematol.

[R20] Pedoeem A, Azoulay-Alfaguter I, Strazza M, Silverman GJ, Mor A (2014). Programmed death-1 pathway in cancer and autoimmunity. Clin Immunol.

[R21] Pardoll DM (2012). The blockade of immune checkpoints in cancer immunotherapy. Nat Rev Cancer.

[R22] Miller PL, Carson TL (2020). Mechanisms and microbial influences on CTLA-4 and PD-1-based
immunotherapy in the treatment of cancer: a narrative review. Gut Pathog.

[R23] Topalian SL, Hodi FS, Brahmer JR, Gettinger SN, Smith DC, McDermott DF, et al (2012). Safety, activity, and immune correlates of anti–PD-1 antibody in
cancer. New Engl J Med.

[R24] Hamid O, Robert C, Daud A, Hodi FS, Hwu WJ, Kefford R, et al (2013). Safety and tumor responses with lambrolizumab (anti-PD-1) in
melanoma. N Engl J Med.

[R25] Szczepanski MJ, Szajnik M, Czystowska M, Mandapathil M, Strauss L, Welsh A, et al (2009). Increased frequency and suppression by regulatory T cells in
patients with acute myelogenous leukemia. Clin Cancer Res.

[R26] Lichtenegger FS, Lorenz R, Gellhaus K, Hiddemann W, Beck B, Subklewe M (2014). Impaired NK cells and increased T regulatory cell numbers during
cytotoxic maintenance therapy in AML. Leuk Res.

[R27] Amarnath S, Mangus CW, Wang JC, Wei F, He A, Kapoor V, et al (2011). The PDL1-PD1 axis converts human TH1 cells into regulatory T
cells. Sci Transl Med.

[R28] Ansell SM, Lesokhin AM, Borrello I, Halwani A, Scott EC, Gutierrez M, et al (2015). PD-1 blockade with nivolumab in relapsed or refractory Hodgkin's
lymphoma. N Engl J Med.

[R29] Wolchok JD, Kluger H, Callahan MK, Postow MA, Rizvi NA, Lesokhin AM, et al (2013). Nivolumab plus ipilimumab in advanced melanoma. N Engl J Med.

[R30] Zhang W, Bai JF, Zuo MX, Cao XX, Chen M, Zhang Y, et al (2016). PD-1 expression on the surface of peripheral blood CD4+ T cell
and its association with the prognosis of patients with diffuse large B-cell
lymphoma. Cancer Med.

[R31] MacFarlane AWt, Jillab M, Plimack ER, Hudes GR, Uzzo RG, Litwin S, et al (2014). PD-1 expression on peripheral blood cells increases with stage in
renal cell carcinoma patients and is rapidly reduced after surgical tumor
resection. Cancer Immunol Res.

[R32] Liersch R, Muller-Tidow C, Berdel WE, Krug U (2014). Prognostic factors for acute myeloid leukaemia in
adults--biological significance and clinical use. Br J Haematol.

[R33] Muenst S, Hoeller S, Dirnhofer S, Tzankov A (2009). Increased programmed death-1+ tumor-infiltrating lymphocytes in
classical Hodgkin lymphoma substantiate reduced overall survival. Hum Pathol.

[R34] Richendollar BG, Pohlman B, Elson P, Hsi ED (2011). Follicular programmed death 1-positive lymphocytes in the tumor
microenvironment are an independent prognostic factor in follicular lymphoma. Hum Pathol.

[R35] Smeltzer JP, Jones JM, Ziesmer SC, Grote DM, Xiu B, Ristow KM, et al (2014). Pattern of CD14+ follicular dendritic cells and PD1+ T cells
independently predicts time to transformation in follicular lymphoma. Clin Cancer Res.

[R36] Yang ZZ, Grote DM, Ziesmer SC, Xiu B, Novak AJ, Ansell SM (2015). PD-1 expression defines two distinct T-cell sub-populations in
follicular lymphoma that differentially impact patient survival. Blood Cancer Journal.

[R37] Carreras J, Lopez-Guillermo A, Roncador G, Villamor N, Colomo L, Martinez A, et al (2009). High numbers of tumor-infiltrating programmed cell death
1-positive regulatory lymphocytes are associated with improved overall
survival in follicular lymphoma. J Clin Oncol.

[R38] Adom D, Dillon SR, Yang J, Liu H, Ramadan A, Kushekhar K, et al (2020). ICOSL+ plasmacytoid dendritic cells as inducer of graft-versus-host disease, responsive to a dual ICOS/CD28 antagonist. Sci Transl Med.

[R39] Saadi MI, Yaghobi R, Karimi MH, Geramizadeh B, Ramzi M, Zakerinia M (2013). Association of the costimulatory molecule gene polymorphisms and
active cytomegalovirus infection in hematopoietic stem cell transplant
patients. Mol Biol Rep.

[R40] Dolen Y, Esendagli G (2013). Myeloid leukemia cells with a B7‐2+ subpopulation provoke Th‐cell
responses and become immuno‐suppressive through the modulation of B7 ligands. Eur J Immunol.

